# Electroacupuncture at Bilateral Zusanli Points (ST36) Protects Intestinal Mucosal Immune Barrier in Sepsis

**DOI:** 10.1155/2015/639412

**Published:** 2015-08-04

**Authors:** Mei-fei Zhu, Xi Xing, Shu Lei, Jian-nong Wu, Ling-cong Wang, Li-quan Huang, Rong-lin Jiang

**Affiliations:** Department of Critical Care Medicine, The First Affiliated Hospital, Zhejiang Chinese Medical University, Hangzhou 310006, China

## Abstract

Sepsis results in high morbidity and mortality. Immunomodulation strategies could be an adjunctive therapy to treat sepsis. Acupuncture has also been used widely for many years in China to treat sepsis. However, the underlying mechanisms are not well-defined. We demonstrated here that EA preconditioning at ST36 obviously ameliorated CLP-induced intestinal injury and high permeability and reduced the mortality of CLP-induced sepsis rats. Moreover, electroacupuncture (EA) pretreatment exerted protective effects on intestinal mucosal immune barrier by increasing the concentration of sIgA and the percentage of CD3+, *γ*/*δ*, and CD4+ T cells and the ratio of CD4+/CD8+ T cells. Although EA at ST36 treatments immediately after closing the abdomen in the CLP procedure with low-frequency or high-frequency could not reduce the mortality of CLP-induced sepsis in rats, these EA treatments could also significantly improve intestinal injury index in rats with sepsis and obviously protected intestinal mucosal immune barrier. In conclusion, our findings demonstrated that EA at ST36 could improve intestinal mucosal immune barrier in sepsis induced by CLP, while the precise mechanism underlying the effects needs to be further elucidated.

## 1. Introduction

Sepsis frequently occurs after trauma, burns, hemorrhage, or abdominal surgery. It can progress to multiorgan failure (MOF). Although new treatment algorithms focusing on rapid administration of broad spectrum antibiotics and aggressive restoration of tissue oxygen delivery have led to decreases in mortality, the death rate is still high (~30%) [1–4]. Effective and safe treatments for this disease are desperately needed.

Sepsis is a complex immune syndrome characterized by an imbalance between pro- and anti-inflammatory mediators systemically released in high amounts (cytokine storm) in response to an infection, while the precise pathophysiologic mechanisms underlying the development of multiorgan failure (MOF) remain elusive [5, 6]. The gut is often described as the motor of MOF because the loss of its integrity is a critical comorbidity factor for patients after HS, as well as trauma, surgery, sepsis, and burn injuries [7, 8]. Therefore, the intestinal mucosal immune system has recently emerged as a potential target in sepsis treatment.

Traditional Chinese medicine (TCM, including Chinese materia medica, acupuncture, and physiotherapy) always attached importance to gastrointestinal function, believing that the stomach and spleen provide the material basis for the acquired constitution. Acupuncture has also been used widely for many years in China to treat sepsis. Recently, acupuncture has been described as a complementary and alternative medicine (CAM) in which filiform needles are inserted at specific points on the body, called acupoints, which can subsequently be stimulated in various ways, such as electroacupuncture (EA) [9]. It has been reported that in rats with CLP sepsis models EA-ST36 reduces serum TNF levels through VN- and catecholamine-dependent mechanisms. Indeed, the treatment with EA at ST36 can decrease levels of proinflammatory cytokine expression (TNF-*α*, IL-1, and IL-6) in a lipopolysaccharide-induced model of acute nephritis, collagen-induced arthritis mouse model, ulcerative colitis rat model, carrageenan-induced mouse model of inflammation, and cerebral ischemia-reperfusion injured rats. Recent studies have shown that the mechanism underlying the effects of this treatment is related to the suppression of the TLR4/NF-*κ*B signaling pathway [10–13].

Although the recent focus has been on the function of EA at ST36, there are a lot of problems to be solved and whether EA at ST36 protects gut barrier is uncertain. The object of this study, based on the rat model of sepsis induced by CLP, was to observe the effects of EA at ST36 on the intestinal mucosal immune barrier.

## 2. Materials and Methods

### 2.1. Animals

Male Sprague Dawley (SD) rats (body weight, 180–220 g) were obtained from the Zhejiang province Experimental Animal Center and housed in the laboratory animal center of Zhejiang Chinese Medical University at 22°C with a 12-hour light/dark cycle. All animals used in the study were housed and cared for in accordance with the Chinese Pharmacological Society Guidelines for Animal Use. The work was approved by the Committee on the Ethics of Animal Experiments of the Zhejiang Chinese Medical University (permit number: 2012-0049). All surgery was performed under sodium pentobarbital anesthesia, and all efforts were made to minimize suffering.

### 2.2. Experimental Sepsis Model by CLP

The rats were subjected to CLP as previously described. Briefly, under aseptic conditions, a 3-cm midline laparotomy was performed to allow the exposure of the cecum and adjoining intestine. The cecum was tightly ligated with a 2.0-silk suture at its base, below the ileocecal valve, and was perforated twice with an 18-gauge needle. The cecum was then gently squeezed to extrude a small amount of feces from the puncture site. The cecum was then returned to the peritoneal cavity and the laparotomy was closed with 3.0-silk sutures. Sham-operated animals underwent the same surgical procedure although the cecum was neither ligated nor punctured. Saline (3 mL/100 g) was administered to all rats intraperitoneally at the end of the procedure. All animals were returned to their cages with free access to food and water.

### 2.3. Acupuncture Treatment Procedure

Two pairs of stainless steel needles (diameter, 0.3 mm; length, 30 mm (Suzhou Medical Supplies Co., Jiangsu, China)) were inserted perpendicularly at a depth of 6 mm into the bilateral Zusanli acupoints (ST36), located 5 mm below and lateral to the anterior tubercle of the tibia. EA stimulation was applied at both bilateral ST36 acupoints, and both output leads from the Programmable Electro-Acupuncture Stimulator (HANS, LH202H, Huawei Co., Beijing, China) were connected to the handles of both needles inserted at ST36 acupoints. EA was applied for 30 min, with an intensity of 2 mA and 2–100 Hz.

### 2.4. Experimental Groups and Protocol

The five groups of animals used in the present study were (1) the sham-operated group (sham, *n* = 20), which underwent a laparotomy; (2) the sepsis group (sepsis, *n* = 20), which underwent CLP; (3) the low-frequency EA group (sepsis + low-frequency EA, *n* = 20), which underwent ST36 acupuncture immediately after closing the abdomen in the CLP procedure and 24 hrs later; (4) the high-frequency EA group (sepsis + high-frequency EA, *n* = 20), which underwent ST36 acupuncture immediately after closing the abdomen in the CLP procedure and 6 hrs, 12 hrs, 18 hrs, and 24 hrs later; and (5) EA preconditioning group (EA + sepsis, *n* = 20), which underwent CLP immediately prior to the application of five days of ST36 acupuncture, once a day.

The rats were kept at a constant environmental temperature of 37°C to maintain body heat following the procedures. At 36 h after the CLP, the rats were reanesthetized, then their abdomens were opened, and ileum was removed for the determination of intestinal mucosal tissue sIgA levels, flow cytometry assay, and histomorphological determination.

### 2.5. Histological Examination

The fixed intestinal mucosal tissue was cut into 3-mm thickness blocks. The tissue blocks were embedded in paraffin and cut into 4 *μ*m slices. After being deparaffinized using xylene and ethanol dilutions and rehydration, the sections were stained with hematoxylin and eosin (H&E, Bogoo, Shanghai, CN) to examine the tissue structure, inflammatory cell infiltration, and necrosis.

### 2.6. sIgA Measurement in Intestinal Mucosa and D-Lactose Measurement in Plasma

Intestinal mucosal tissue sIgA level and D-Lactose measurement in plasma were determined by using ELISA Kit (Boster biological Inc., Wuhan, China) following the manufacturer's protocol.

### 2.7. Flow Cytometry Assay

Follicle-free mucosa destined for mononuclear cells (MC) isolation was cut into smaller pieces of approximately 1 mm^2^. These mucosa pieces were incubated separately for 30 min at 37°C in calcium- and magnesium-free Hanks's balanced salt solution, including 1 mM ethylenediamine tetra-acetic acid (Sigma-Aldrich, Shanghai, China) to remove both epithelium and intraepithelial lymphocytes. After washing, the follicle-free mucosal tissue was disrupted mechanically into smaller pieces of approximately 1-2 mm^3^ and incubated in RPMI-1640 medium supplemented with 30 mM HEPES, 10% fetal calf serum (FCS), 100 U/mL penicillin, 100 *μ*g/mL streptomycin, 50 *μ*g/mL gentamicin, and 2.5 *μ*g/mL amphotericin (all from MCE, Shanghai, China), 0.1% collagenase type IV (Sigma-Aldrich, Shanghai, China), 0.05% DNase I (Roche Diagnostics, Shanghai, China), and 1 *μ*L/mL 2-mercaptoethanol (Sigma-Aldrich, Shanghai, China) at 37°C in humidified 5% CO_2_ atmosphere for 1.5~2 hrs with continuous agitation and vigorous vortex every 15 min. Remaining tissue aggregates were removed by a 70 *μ*m nylon cell strainer. The resulting cell suspension was centrifuged at 500 g for 25 min in a 30% isotonic Percoll solution (Borunlaite Sci & Tech Co., Ltd., Beijing, China). The supernatant containing epithelial cells and debris was discarded; the cell pellet was washed and resuspended in RPMI-1640 medium. The MC fraction additionally underwent Ficoll (Borunlaite Sci & Tech Co., Ltd., Beijing, China) density centrifugation to remove erythrocytes. More than 96% of the isolated cell population was MC.

For cytofluorometric analyses cells were preincubated in PBS and stained for 30 min at 4°C using saturating concentrations of the following antibodies: CD3-FITC+*γ*/*δ* T-PE and CD4-FITC+CD8-PE antibody (Becton, Dickinson and Company, Franklin Lakes, NJ, USA). Cells were analyzed in a Becton-Dickinson LSRII cytometer using FACS Diva software (Becton Dickinson).

## 3. Result

### 3.1. Survival Study

**Figure 1:**
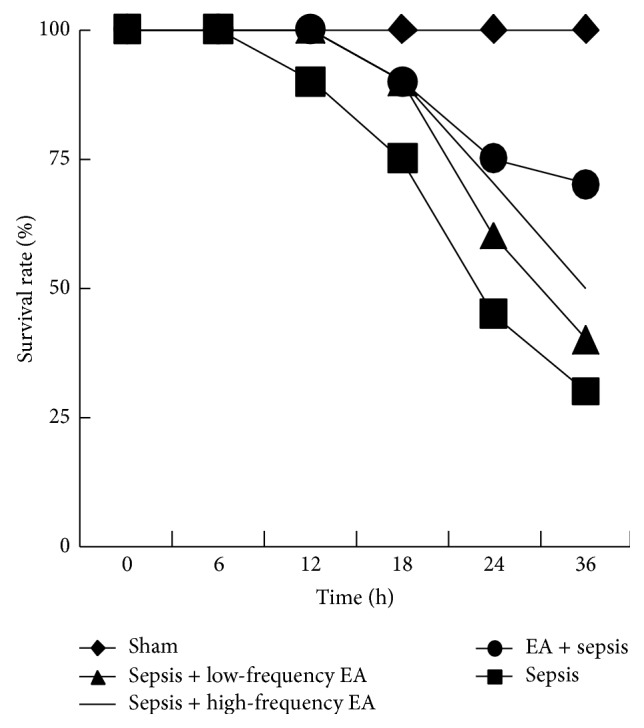
EA preconditioning therapy improved the survival rate in a rat model of sepsis induced by CLP. The percentage survival in 72 hours after surgery is shown.

Survival rate of animals was determined. In sepsis, treatment and prevention groups showed significantly lower survival rate compared to sham group. Although low-frequency EA and high-frequency EA treatment groups had more survival rate than sepsis group (50% and 37% versus 26%), the difference between sepsis and these groups was not significant. In EA + sepsis group, EA preconditioning therapy improved the survival rate significantly (70%) (Figure 1).

### 3.2. EA at ST36 Ameliorates CLP-Induced Intestinal Injury

**Figure 2:**
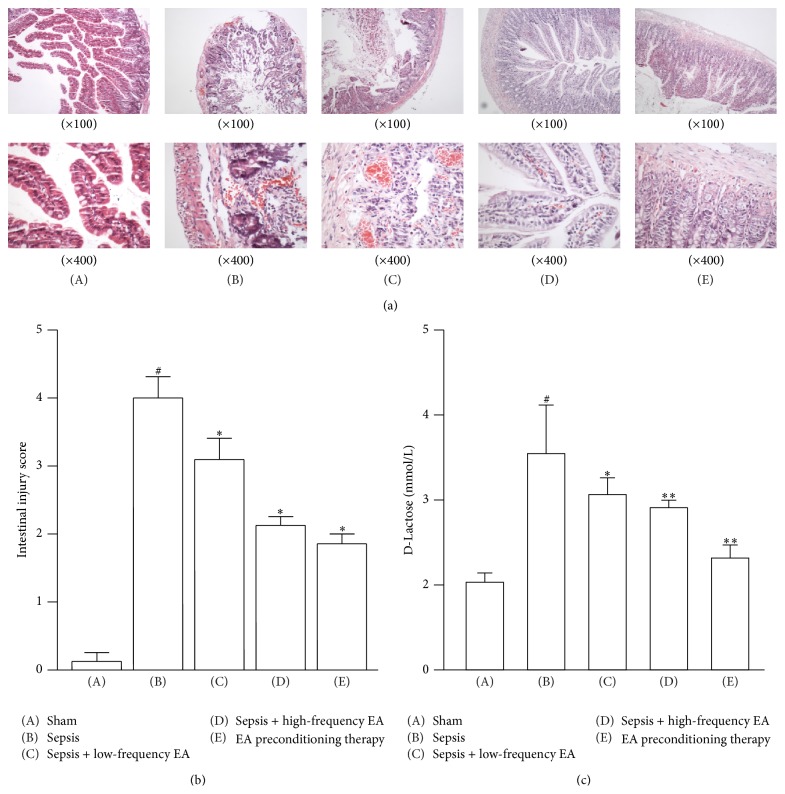
EA at ST36 ameliorates CLP-induced intestinal injury. (a) Ileums were harvested 36 h after CLP for histopathologic examination using H&E staining. Representative images from five animals per group were shown. (b) Semiquantitative analysis of histological samples of ileum showed that EA at ST36 significantly decreased CLP-induced intestinal injury. (c) Effects of EA on the concentration of D-Lactose in the serum. Data were presented as means ± SD (*n* = 5) and ^#^ *p* < 0.01, ^∗^ *p* < 0.05, and ^∗∗^ *p* < 0.01 difference with sham or sepsis group.

The degree of intestinal injury sepsis was evaluated in CLP rats treated with or without EA at ST36. As shown in Figure 2(a), histological analysis showed that the ileum from sham mice had the normal architecture of the intestinal epithelium and wall, while CLP induced severe edema and sloughing of the villous tips, as well as infiltration of inflammatory cells into the mucosa. Semiquantitative analysis of histological samples of ileum showed that the intestinal injury score in the septic mice was significantly increased compared with that in the sham group. Administration of EA at ST36 significantly decreased CLP-induced intestinal injury (Figure 2(b)).

We also detected the circulating D-Lactose that can be considered an indirect indication of intestinal permeability. As shown in Figure 2(c), the concentration of circulating D-Lactose was increased significantly in the septic mice. Treatment with high-frequency EA and EA pretreatment could significantly inhibit the increase of circulating D-Lactose induced by CLP.

### 3.3. Changes of sIgA Content in Intestinal Mucosa Cells

**Figure 3:**
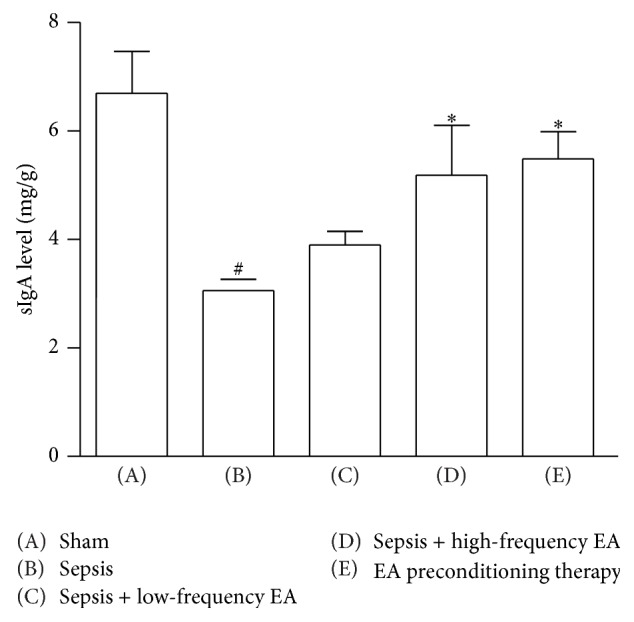
Changes of sIgA content in intestinal mucosa cells. Data were presented as means ± SD (*n* = 5) and ^#^ *p* < 0.01 and ^∗^ *p* < 0.05 difference with sham or sepsis group.

The sIgA concentrations in intestinal mucosa reduced significantly 36 hrs after CLP was developed, while the intestinal mucosal tissue sIgA levels in high-frequency EA group and EA pretreatment group were increased significantly (Figure 3).

### 3.4. Percentage of CD3+, CD4+, and CD8+ T Lymphocytes in Intestinal Mucosa

**Figure 4:**
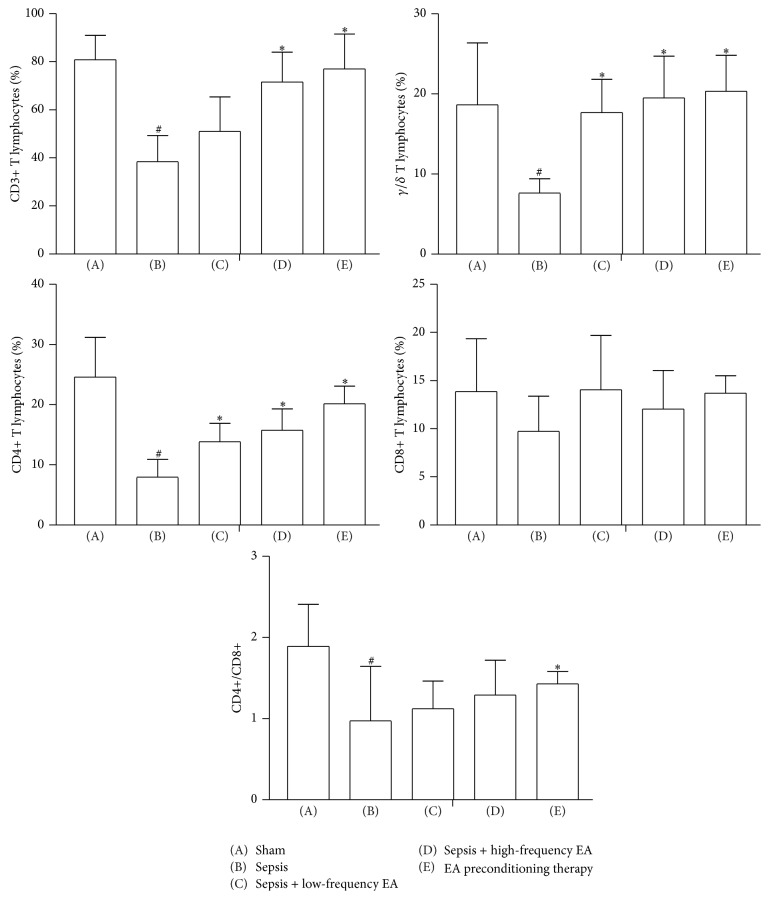
Effects of EA on percentage of CD3+, CD4+, and CD8+ T lymphocytes in intestinal mucosa. Data were presented as means ± SD (*n* = 5) and ^#^ *p* < 0.01 and ^∗^ *p* < 0.05 difference with sham or sepsis group.

As shown in Figure 4, compared with sham group, CD3+, *γ*/*δ*, CD4+, and CD4+/CD8+ T lymphocytes were significantly decreased from (80.75 ± 10.24)%, (18.64 ± 7.73)%, (24.59 ± 6.60)%, and (1.89 ± 0.52) to (38.38 ± 10.90)%, (7.62 ± 1.79)%, (7.95 ± 2.95)%, and (0.97 ± 0.67) in the sepsis group. Low-frequency EA could increase the percentage of CD3+, *γ*/*δ*, and CD4+ T cells as compared with model group. EA preconditioning significantly increased the percentage of CD3+, *γ*/*δ*, and CD4+ T cells and the ratio of CD4+/CD8+ T cells to (77.08 ± 14.43)%, (20.33 ± 4.84)%, (20.14 ± 2.94)%, and (1.43 ± 0.15). However, no difference of the percentage of CD8+ T cells was observed between five groups.

## 4. Discussion

In the present studies, we showed that the intestinal mucosal immune barrier was seriously damaged in a rat model of sepsis induced by CLP. EA preconditioning at ST36 obviously ameliorated CLP-induced intestinal injury and high permeability and exerted protective effects on intestinal mucosal immune barrier; EA preconditioning significantly increased the percentage of CD3+, *γ*/*δ*, and CD4+ T cells and the ratio of CD4+/CD8+ T cells and ultimately reduced the mortality of CLP-induced sepsis in rats. Although EA treatments at ST36 with low-frequency and high-frequency could not reduce the mortality of CLP-induced sepsis in rats, these EA treatments could also significantly improve intestinal injury index in rats with sepsis and obviously protected intestinal mucosal immune barrier.

The gastrointestinal tract is an organ of digestion and absorption. In recent years, the gastrointestinal tract has assumed more importance in the management of the septic patient in the intensive care unit [14–16]. It is now recognized that the small intestine and colon make important contributions to the maintenance of hypermetabolism in sepsis. Owing to the increased intestinal permeability with gut barrier injury, the bacteria and lipopolysaccharide (LPS) can enter the systemic circulation through the portal vein and the mesenteric lymph and result in sepsis and multiple organ dysfunction syndromes (MODS) [17]. Therefore, the intestinal tract is regarded as “initiator” of MODS and supporting general immune function and maintaining the structure and function of the gastrointestinal tract were possible therapeutic strategies for sepsis [14, 18].

Based on semiquantitative histological examination and the mucosal damage index, we found that the ileum from the mice of sham group had the normal architecture of the intestinal epithelium and wall, while CLP induced severe edema and sloughing of the villous tips, as well as infiltration of inflammatory cells into the mucosa. The intestinal injury score in the septic mice was significantly increased. Administration of EA at ST36 significantly decreased CLP-induced intestinal injury. It suggested that EA at ST36 improved the restitution and mechanical barriers of intestinal mucosa in CLP-induced sepsis. Moreover, plasma D-lactate, produced by intestinal bacteria, was developed as a biomarker of intestinal high permeability [19]. Here, we showed that the concentration of circulating D-Lactose was increased significantly in the septic mice. Treatment with high-frequency EA and EA pretreatment could significantly inhibit the increase of circulating D-Lactose induced by CLP. It indicated that EA could protect the intestinal mucosa epithelial cells and maintain the integrity of gut mucosal barrier.

The gut mucosal barrier comprises both immunological and nonimmunological protective components, with the former being divided into local and systemic components and the latter comprising mechanical and chemical barriers. The sIgA content and percentage of T lymphocytes in intestinal mucosa is important to local intestinal mucosal immune barrier [20]. In this study, both of high-frequency EA treatment and EA preconditioning could increase the intestinal mucosal sIgA concentration. Furthermore, EA preconditioning significantly increased the percentage of CD3+, *γ*/*δ*, and CD4+ T cells and the ratio of CD4+/CD8+ T cells.

These results supported that EA treatments at ST36 with low-frequency, high-frequency, and preconditioning can protect the intestinal barrier to different degrees, among which EA preconditioning exerts positive effects on immune barrier and eventually decreases the mortality of sepsis.
